# *Meta*-Topolin as an Aromatic Cytokinin for *In Vitro* Propagation of *Thymus vulgaris* L.

**DOI:** 10.3390/plants14233567

**Published:** 2025-11-22

**Authors:** Mologadi B. Mabotja, Adeyemi O. Aremu, Karel Doležal, Oziniel Ruzvidzo, Stephen O. Amoo

**Affiliations:** 1 Department of Botany, School of Biological Sciences, Faculty of Natural and Agricultural Sciences, North-West University, Private Bag X2046, Mmabatho 2745, South Africa; mabotjamb@arc.agric.za (M.B.M.); oziniel.ruzvidzo@nwu.ac.za (O.R.); 2 Agricultural Research Council—Vegetables, Industrial, and Medicinal Plants, Private Bag X293, Pretoria 0001, South Africa; 3 Indigenous Knowledge Systems Centre, Faculty of Natural and Agricultural Sciences, North-West University, Private Bag X2046, Mmabatho 2745, South Africa; oladapo.aremu@nwu.ac.za; 4 Laboratory of Growth Regulators, and Institute of Experimental Botany AS CR, Šlechtitelů 27, CZ-783 71 Olomouc, Czech Republic; karel.dolezal@upol.cz; 5 Department of Chemical Biology, Faculty of Science, Palacký University, Šlechtitelů 27, CZ-783 71 Olomouc, Czech Republic; 6 Unit for Environmental Sciences and Management, Faculty of Natural and Agricultural Sciences, North-West University, Potchefstroom 2531, South Africa

**Keywords:** auxin, Lamiaceae, *meta*-topolin, micropropagation, thyme

## Abstract

Effective in vitro propagation of medicinal and aromatic plants such as *Thymus vulgaris* is often limited by the choice of optimal cytokinin sources, which are critical for successful shoot proliferation and overall regeneration. Since their discovery, topolins have been recognized as alternatives to traditional cytokinins (CKs) such as benzyladenine (BA) and kinetin (Kin) in plant tissue culture (PTC). This study investigated the influence of three aromatic CKs (*meta*-topolin (*m*T), BA, and Kin), on shoot proliferation of thyme, with the goal of improving current PTC protocols. Of all the tested treatments, the highest shoot proliferation (7.25 ± 0.72 shoots per nodal explant) was observed in the treatment with 1 µM *m*T, superior to BA and Kin. Increasing *m*T and BA concentrations from 1 to 15 µM had an inverse effect on shoot production. The addition of indole-3-butyric acid (IBA) at concentrations ranging from 1.0 to 5.0 µM in combination with 0.5 and 1 µM *m*T did not increase the mean shoot numbers per explant. Regenerated shoots exhibited a strong propensity for root development even in the absence of plant growth regulators with 100% survival ex vitro. This study demonstrated that *m*T is an effective, sustainable and cost-effective alternative to traditional CKs for in vitro propagation of *T. vulgaris*, achieving an improved shoot proliferation with 1 μM *m*T application.

## 1. Introduction

The effectiveness of micropropagation efforts depends on a range of complex chemical and physical factors. Among these, plant growth regulators (PGRs) serve as an essential chemical factor that controls numerous physiological and developmental activities throughout the micropropagation process [1]. PGRs such as cytokinins (CKs) and auxins are frequently incorporated into culture media with the intention of regulating diverse physiological reactions in vitro, thereby facilitating the generation of tissues (e.g., callus), organs (e.g., shoots and roots), or whole plants [2].

*Thymus vulgaris* L, commonly known as thyme, is an aromatic herbaceous plant of the Lamiaceae family [3]. It is a popular species of the genus *Thymus* L., which is a taxonomic and systematically complex genus with over 220 plant species that exhibit significant polymorphism not only in morphological characteristics but also in ethereal oil composition [4]. Native to the Mediterranean region, including Southern Europe, Asia and parts of North Africa, *T*. *vulgaris* is the most cultivated species of the genus *Thymus* [5]. Due to its valuable medicinal and aromatic properties, thyme is of considerable economic and therapeutic significance, commonly used in traditional medicine, with applications in the pharmaceutical, cosmetic, food, and feed industries [6]. Traditionally, it was used across cultures for respiratory ailments, wound healing, and digestive issues, owing to its antiseptic properties [7,8,9,10]. Today, its essential oils rich in thymol and carvacrol are widely applied in pharmaceuticals for antimicrobial and anti-inflammatory effects, in the food industry as a natural preservative and flavor enhancer, and in cosmetics and organic farming for their antiseptic and pesticidal qualities [8,11,12,13,14,15].

Micropropagation of *T. vulgaris* offers a promising alternative to conventional propagation methods, particularly in overcoming challenges related to seasonal variability, genetic erosion, and low multiplication rates. However, the literature reveals divergent outcomes in micropropagation protocols, often attributed to differences in physiological and developmental traits among genotypes, and media composition [6]. For instance, Ana-Maria and Ramona [16] demonstrated that Murashige and Skoog (MS) [17] medium devoid of phytohormones yielded the highest shoot multiplication rate and shoot length, while Furmanowa and Olszowska [18] reported successful regeneration using Nitsch and Nitsch (NN) [19] medium supplemented with kinetin (Kin) and auxins such as indole-3-butyric acid (IBA) and 1-naphthaleneacetic acid (NAA). These findings underscore the necessity of optimizing hormonal combinations tailored to specific genotypes and developmental stages. Cytokinins such as benzyladenine (BA), Kin, and 2-isopentenyladenine (2iP) have been widely studied for the micropropagation of *T*. *vulgaris* [20,21]. While BA is frequently reported for shoot induction, its use is often associated with drawbacks including shorter shoots, hyperhydricity, reduced shoot numbers, and tissue browning [6,22,23]. Kulpa et al. [22] reported that 2iP at 5 mg/l produced the highest shoot proliferation compared to BA and Kin. However, contrasting findings by Ana-Maria and Ramona [16] showed that the control medium without growth regulators yielded the best morphogenetic response, while supplementation with BA, Kin, or 2iP inhibited microshoot growth, with inhibitory effects increasing at higher concentrations.

It has been demonstrated that topolins can boost shoot proliferation, preserve histogenic stability, enhance rooting efficiency, and reduce various physiological disorders during micropropagation [24,25]. Moreover, a recent study on *Melissa officinalis*, another species belonging to the Lamiaceae family, revealed that media supplementation with 5 µM *m*T significantly improved shoot proliferation compared to BA and Kin [26]. These outcomes suggest that *m*T may offer similar benefits in *T. vulgaris*. Traditional CKs such as BA and Kin, although widely used in thyme tissue culture, have been associated with several limitations including hyperhydricity [22,23], shoot-tip necrosis [20], reduced shoot length [16,22], and poor rooting efficiency [27]. While the advantageous effects of *m*T have been reported in various in vitro propagated plant species [24,26], their specific influence on in vitro propagated thyme has yet to be investigated. The superior performance of *m*T is hypothesized to stem from its high affinity for the histidine kinase AHK3 CK receptor, which promotes shoot initiation and delays senescence, as well as its enhanced metabolic stability due to reversible *O*-glucosylation [28,29]. Given the economic and therapeutic importance of thyme, there is a compelling need to refine and optimize existing plant tissue culture (PTC) protocols. Therefore, the present research sought to evaluate the influence of three CKs (*m*T in comparison to BA and Kin) applied independently and *m*T in combination with IBA in the micropropagation of thyme, with the goal of improving current PTC protocols.

## 2. Results and Discussion

### 2.1. Influence of Cytokinin Type and Concentration on Shoot Proliferation

**Figure 1:**
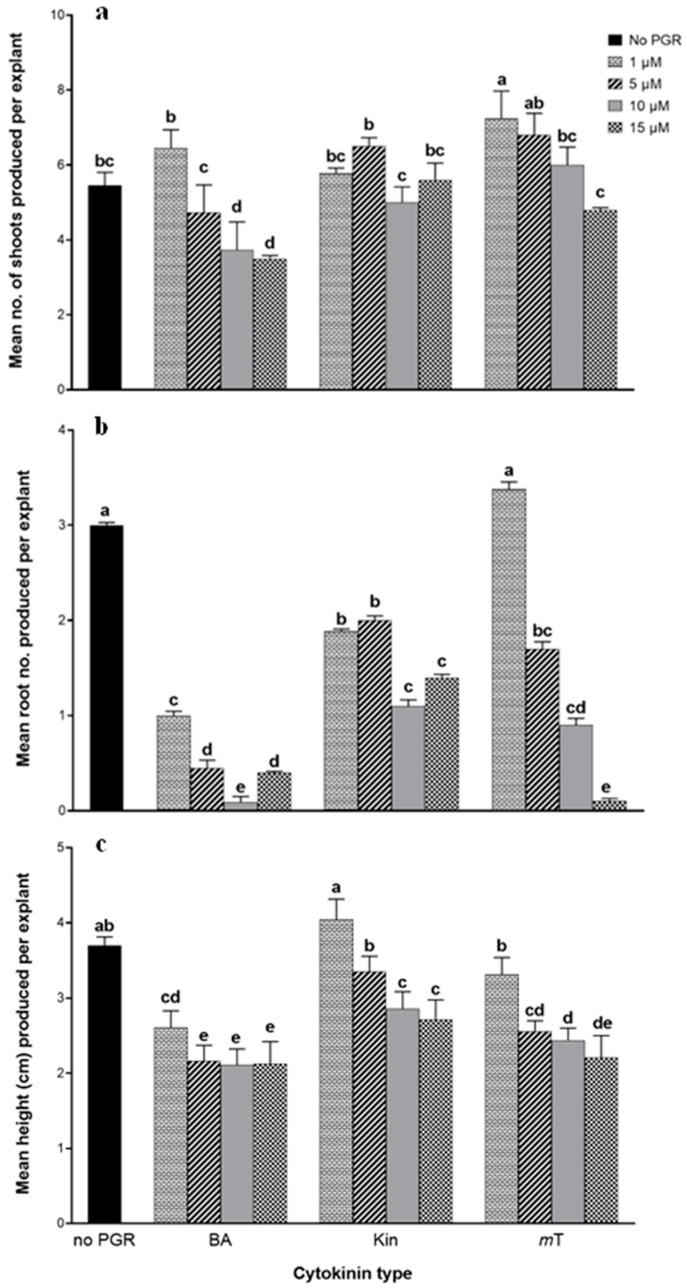
Effect of cytokinins (6-benzyladenine (BA, kinetin (Kin), and *meta*-topolin (*m*T)) on shoot proliferation after 6 weeks of culture: (**a**) mean shoot number; (**b**) mean root number; (**c**) mean height of regenerated shoots per explant (cm). Bars with different letter(s) differ significantly by Duncan’s multiple range test (*p* = 0.05). Values are mean ± standard error, *n* = 15.

**Figure 2:**
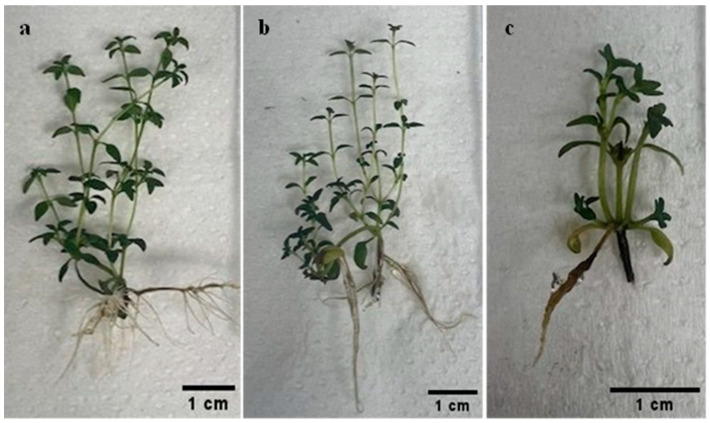
Effect of cytokinins on shoot proliferation after 6 weeks of culture: (**a**) control without plant growth regulator; (**b**) medium with 1 µM *m*T; (**c**) medium with 15 µM BA.

Figure 1 shows the effect of CK type and concentrations on shoot proliferation of *T. vulgaris* after 6 weeks of culture The highest shoot proliferation (7.25 ± 0.72 shoots per nodal explant) was obtained with 1 µM *m*T treatment (Figure 2), which was not statistically different (*p* = 0.05) from the treatment with 5 µM *m*T with a mean shoot number of 6.80 ± 0.58 per explant. The mean shoot numbers recorded in the treatments with 1 µM BA (6.45 ± 0.49), 1 µM (5.78 ± 0.14), 5 µM (6.5 ± 0.23), 15 µM (5.60 ± 0.45) Kin and 10 µM *m*T (6.00 ± 0.48) were not statistically different (*p* = 0.05) from that of the control treatment (5.40 ± 0.59). The lowest mean shoot number (3.50 ± 0.09) was observed in 15 µM BA treatment (Figure 2c).

With regard to the mean root number produced per explant, the highest number of roots (3.38 ± 0.08) was recorded in the treatment with 1 µM *m*T, which was not statistically different (*p* = 0.05) from roots produced in the PGR-free treatment (3.00 ± 0.03), while the lowest numbers of roots (0.09 ± 0.06 and 0.10 ± 0.03) were recorded in 10 µM BA and 15 µM *m*T, respectively. Shoots grown on treatments with 1 µM Kin and PGR-free treatment had the highest heights per explant, i.e., 4.04 ± 0.27 cm and 3.70 ± 0.11 cm, respectively.

A prior study indicated that the optimal shoot growth of *T*. *vulgaris* occurred on media enriched with 2 mg/l BA, in comparison to treatments with Kin, thidiazuron (TDZ), and 6-(γ,γ-dimethylallylamino)purine (2iP) [21]. The study revealed a favorable correlation between increasing CK concentration (0.5–2 mg/l) and shoot production [21]. However, this was not observed in the present investigation, particularly with the increasing concentrations of BA and *m*T. In this study, *m*T at 1 μM produced 7.25 ± 0.72 shoots per nodal explant, which is higher than values reported for other cytokinins in previous studies [22]. Ana-Maria and Ramona [16] reported the highest shoot numbers of 3.4, 2.7, and 2.7 for BA, 2iP, and Kin at 1 mg/L, respectively, and further increases in cytokinin concentrations led to a decline in shoot proliferation. Kulpa et al. [22] observed up to 16 shoots with 2iP and 10 shoots with BA treatments, each at 5 mg/l per jar containing 5 nodal explants (translating to a maximum of 3 shoots per explant), and regenerants from BA treatments were associated with hyperhydricity symptoms. A possible explanation for this difference may lie in genotype-specific responses to the cytokinins tested, as different cultivars or seed sources of *T. vulgaris* can exhibit variable hormonal sensitivities [20,30]. Additionally, differences in culture conditions such as light intensity, vessel type, medium composition, and explant physiological status may influence cytokinin uptake and metabolism [31,32].

Ozudogru et al. [30] found that MS medium supplemented with 1 mg/l Kin favored regeneration and multiple shoot formation compared to BA, TDZ, or 2iP. Similarly, Ana-Maria and Ramona [16] observed that Kin performed better than other cytokinins tested; however, their study also showed that the highest shoot multiplication occurred on PGR-free MS medium. While there is no documentation about the application of *m*T in the shoot multiplication of *T. vulgaris*, its efficacy in achieving substantial shoot proliferation, in comparison to BA and Kin, however, has been reported in other *Lamiaceae* species including *Salvia sclarea* L. and *M. officinalis* [26,33].

The process of shoot regeneration and proliferation in micropropagation is influenced by the type and concentration of PGRs used, with CKs playing a particularly vital role due to their significance in promoting cell division and organ formation [34]. CKs promote shoot proliferation primarily by stimulating cell division and the differentiation of shoot buds from existing meristematic tissues [35]. CKs activate specific genes involved in cell proliferation and organogenesis, leading to the development of multiple shoots [36]. Additionally, CKs influence the balance of plant hormones, particularly auxins, shifting the growth response towards shoot proliferation rather than root formation [36].

The visible morphological differences among plantlets shown in Figure 2 reflect the influence of cytokinin type and concentration on shoot structure and tissue physiology. Cultures grown on 1 µM *m*T exhibited compact shoots with well-developed leaves, whereas those on 15 µM BA displayed symptoms of hyperhydricity with brittle leaves and stunted growth. These variations are consistent with previous reports that supra-optimal BA levels can lead to symptoms of hyperhydricity [22,23]. The superior rooting performance observed in shoots from 1 µM *m*T treatment compared to BA and Kin can be attributed to *m*T unique structural and metabolic properties [24]. Unlike BA, which inhibits rooting in some species due to accumulation of toxic *N*-glucosides in basal tissues, *m*T undergoes reversible *O*-glucosylation, forming storage forms that maintain hormonal balance and reduce physiological stress [37,38]. This property likely facilitated normal water relations and cell wall development, resulting in robust roots. Previous studies have shown that low concentrations of *m*T (around 1–1.33 µM) promote rooting and acclimatization, while higher concentrations exert inhibitory effects [39,40]. Furthermore, the healthy morphology and strong rooting capacity under *m*T treatment correlate with improved acclimatization success, as reported in *Aloe polyphylla* and *Spathiphyllum floribundum* [38,41].

Based on the observed effect after 6 weeks of culturing, there was an inverse relationship in shoot production with increasing *m*T and BA concentrations from 1 to 15 µM. This decline in shoot proliferation may be attributed to the disruption of the total hormonal equilibrium, encompassing both endogenous and exogenously applied CKs due to supra-optimal CK levels. Excessive exogenous CKs can lead to hormonal toxicity, which interferes with the finely tuned hormonal crosstalk required for effective organogenesis [37,42,43].

The significant increase in shoot proliferation observed with *m*T in this study can be linked to its distinctive chemical structure and physiological properties. Unlike other CKs such as BA, *m*T features an extra hydroxyl group attached to its benzyl ring. This additional hydroxyl group in its side chain promotes the formation of *O*-glucoside metabolites, which are reversible forms that enhance its stability and availability within PTC systems [29]. This contrasts with BA, which is prone to irreversible *N*9-glucosylation, often leading to reduced activity and increased toxicity [44,45].

The superior performance of *m*T at low concentrations can be further attributed to its strong activation of the AHK3 and CRE1/AHK4 CK receptors, which are central to shoot initiation and leaf longevity [28]. It is possible that the CK receptors in thyme have a higher binding affinity for *m*T compared to BA and Kin, enabling *m*T to more effectively activate CK signaling pathways at the lower concentrations and thereby promoting enhanced shoot proliferation. These properties likely contribute to the enhanced shoot proliferation observed at lower *m*T concentrations.

### 2.2. Influence of the Combination of mT and Indole-Butyric Acid on Shoot Proliferation

**Figure 3:**
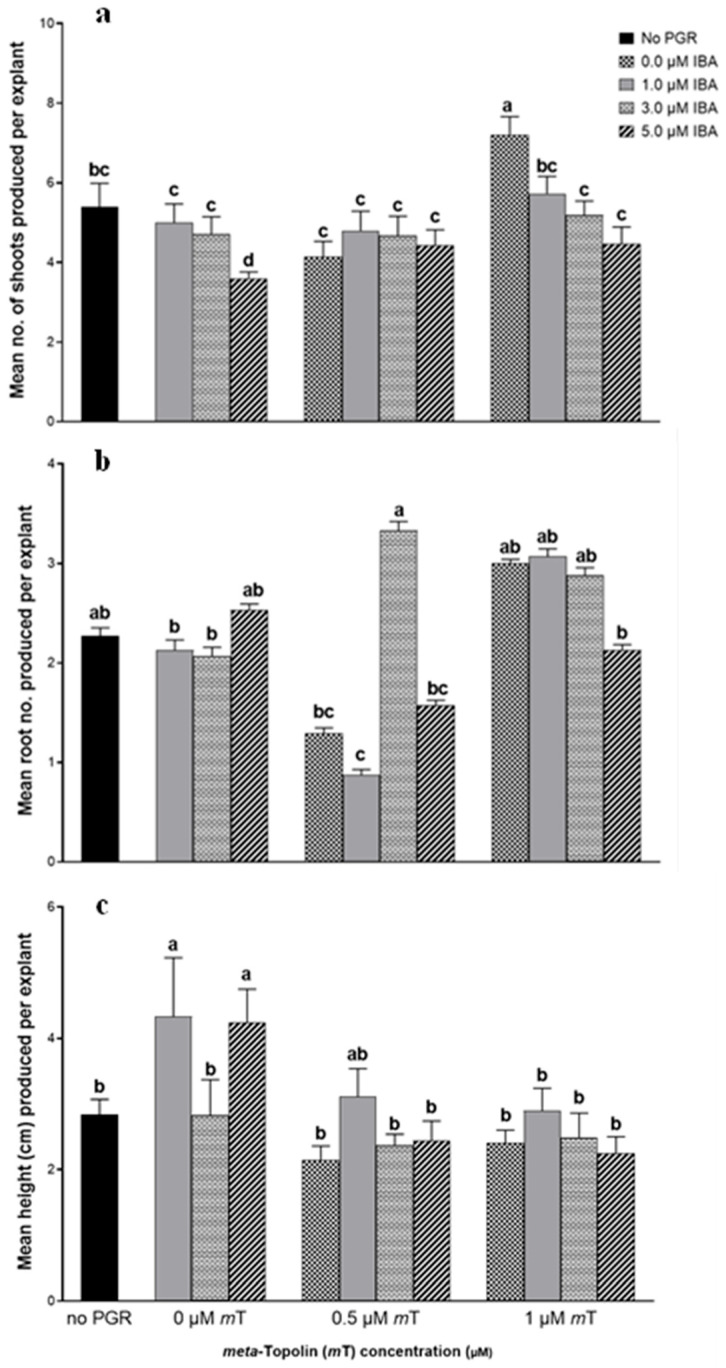
Effect of the combination of *meta*-topolin (*m*T) and indole-butyric acid (IBA) on shoot proliferation after 6 weeks of culture: (**a**) mean shoot number; (**b**) mean root number; (**c**) mean height (cm) produced per explant. Bars with different letter(s) differ significantly by Duncan’s multiple range test (*p* = 0.05). Values are mean ± standard error, *n* = 15.

**Figure 4:**
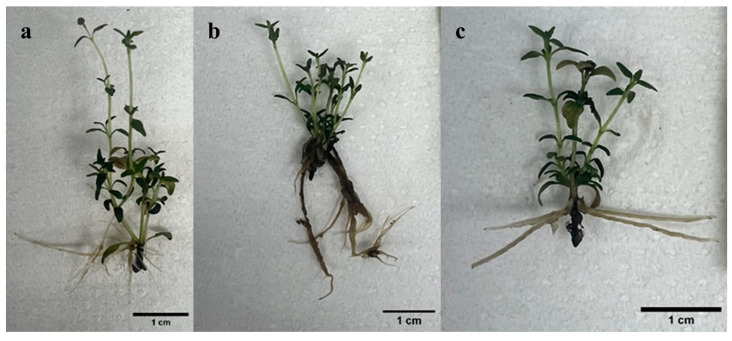
Effect of the combination of *meta*-topolin (*m*T) and indole-butyric acid (IBA) on shoot proliferation after 6 weeks of culture: (**a**) Medium containing 1 µM *m*T and 1 µM IBA. (**b**) Medium containing 1 µM *m*T and 3 µM IBA. (**c**) Medium containing 1 µM *m*T and 5 µM IBA.

Auxins (such as IBA) are known to modulate CK biosynthesis and physiological actions which may result in additive, synergistic, or antagonistic interaction effect during organogenesis, for example [46,47]. As illustrated in Figure 3, the addition of IBA at the tested concentrations, either alone or in combination with 0.5 and 1 µM *m*T did not result in any increase in mean shoot number per explant after 6 weeks of culture. The highest shoot number per explant (7.21 ± 0.45) was recorded in the treatment with 1 µM *m*T. An increase in IBA concentration resulted in a decrease in shoot number when tested alone or in combination with 1 µM *m*T (Figure 4). The lowest shoot number per explant was observed in treatment with 5 µM IBA.

The supplementation of media with either 1 and 5 µM IBA resulted in the highest mean height of 4.33 ± 0.90 cm and 4.04 ± 0.27 cm, respectively. The treatment with 0.5 µM *m*T yielded the highest mean number of roots (3.33 ± 0.91) per explant. However, this was not statistically different (*p* = 0.05) to that recorded in PGR-free treatment (2.27 ± 0.83) and treatments with 5 µM IBA (2.53 ± 0.62), 1 µM *m*T (3.00 ± 0.41), 1 µM *m*T + 1 µM IBA (3.07 ± 0.78), and 1 µM *m*T + 3 µM IBA (2.88 ± 0.78).

These findings suggest that applying exogenous IBA is not necessary for inducing shoots or promoting shoot proliferation from nodal explants in *T vulgaris*, exhibiting an antagonistic response on shoot multiplication with the inclusion of IBA. While synergistic effects between *m*T and IBA on shoot proliferation have been reported in species such as *Coleonema album* [48], *Huernia hystrix* [2] and *Ansellia africana* [49], our results indicate that *T. vulgaris* responds differently, likely due to species-specific hormonal sensitivity.

The interaction between auxins and CKs is a well-documented regulatory mechanism in PTC, where the balance between these hormones determines organogenic outcomes [50]. CKs promote shoot formation, while auxins such as IBA typically favor root induction and reinforce apical dominance, thereby suppressing lateral bud outgrowth and shoot proliferation [51,52]. Under the tested conditions, the addition of IBA either alone or in combination with *m*T did not enhance shoot proliferation and instead showed a concentration-dependent inhibitory effect. This antagonism is consistent with auxin–CK crosstalk models, where elevated auxin levels can suppress CK-induced shoot development by modulating gene expression and hormonal signaling pathways [53]. Moreover, IBA is known to be converted to IAA in the peroxisome, and excessive auxin levels can disrupt natural hormonal homeostasis, leading to reduced regeneration efficiency or altered organogenic responses [54,55].

As indicated by Farman [56], the role of auxins in plant growth can vary, acting as either promoters or suppressors, depending on the specific concentration administered. Our findings reinforce this concept and highlight the importance of optimizing auxin levels in culture media. The inherent rooting capacity of *T. vulgaris* suggests reliance on its endogenous auxin pool, reducing the necessity for external auxin supplementation during the shoot multiplication phase.

### 2.3. In Vitro Root Induction and Acclimatization

Shoots cultured on half-strength MS medium significantly (*p* = 0.05) produced more shoots (8.43 ± 0.54) and roots (10.75 ± 0.44) per shoot (Table 1, Figure S1) compared to those on full-strength MS medium, which yielded 6.00 ± 0.37 shoots and 7.14 ± 0.39 roots per shoot. In general, thyme exhibited a strong propensity for root development even in the absence of PGRs. This observation is corroborated by multiple studies, which reported higher rooting frequencies of thyme in PGR-free media compared to media supplemented with exogenous PGRs [16,27,30]. The enhanced rooting in PGR-free conditions may be attributed to the plant’s innate hormonal balance and endogenous auxin levels, which are sufficient to stimulate root initiation and elongation [55].

**Figure 5:**
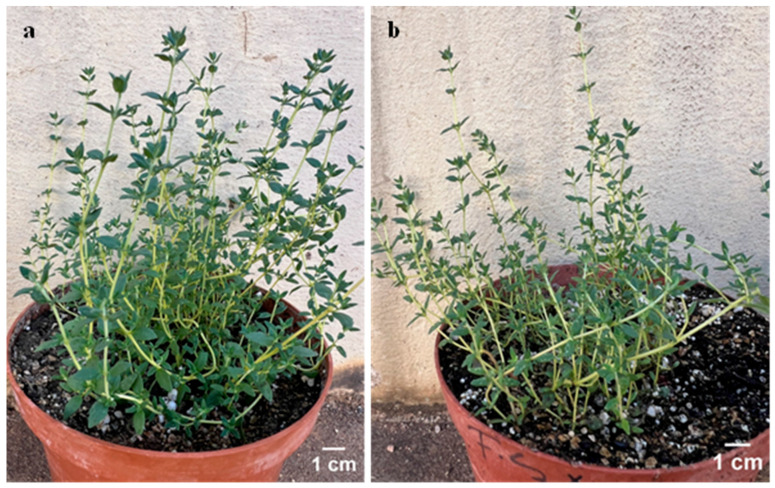
Morphological appearance of acclimatized *Thymus vulgaris* (**a**) regenerated plantlets rooted on half-strength Murashige and Skoog (MS) medium; (**b**) regenerated plantlets rooted on full-strength MS medium.

All rooted regenerants from each treatment were successfully acclimatized using a 1:1 (*v*/*v*) mixture of hygromix and sand, achieving a 100% survival rate (Table 2) with no physical morphological difference (Figure 5). In addition, no differences were observed with the mean shoot height. In many of the previous in vitro studies on micropropagation of thyme, peat was commonly employed as a growth substrate during acclimatization. The reviewed studies reported survival rates ranging from 73 to 96% [16,23,30,57]. With a mixture of soil and peat (1:1), El-Banna [21] was able to achieve a 100% survival rate. The relative high survival rates achieved in this current study suggest that a combination of hygromix and river sand created a well-drained yet moisture-retentive substrate that supported healthy root growth and minimizes water stress. This may be attributable to the porous nature of these substrates [58].

In addition to the substrate selection, the high humidity maintained by misting during the initial critical phase may also have played a role in reducing transpiration and water loss, stabilizing the plant’s physiological functions during the transition from in vitro to ex vitro conditions [59], ultimately leading to a favorable microenvironment that facilitates successful adaptation, leading to a higher survival rate.

## 3. Materials and Methods

### 3.1. Explant Source, Decontamination, and Bulking

Seeds of *T*. *vulgaris* were obtained from Jekka’s Herb Farm in Bristol, UK. According to preliminary research, the seeds were first rinsed with tap water, and submerged in 70% ethanol for 2 min. Subsequently, the seeds were soaked for 1 min in a 3.5% (*v*/*v*) sodium hypochlorite solution containing 1 mL of Tween 20, followed by rinsing with distilled water. After surface sterilization, the seeds were germinated on sterile, 1/10th strength MS medium (pH 5.8), which was pre-sterilized by autoclaving for 20 min at 103 kPa and 121 °C. The germination process took place in a growth room maintained at 25 ± 2 °C under a 16 h light/8 h dark photoperiod, with a light intensity of 60 ± 3 µmol m^−2^ s^−1^ over a period of 6 weeks.

Following germination, the nodal explants were placed in 250 mL CultureJar™ G9 glass culture containers, each sealed with an autoclavable polypropylene lid. Each jar held 30 mL of sterile, full-strength MS medium. The medium’s pH was adjusted to 5.8 prior to solidification with 0.8% (*w*/*v*) bacteriological agar (Oxoid Ltd., Basingstoke, Hampshire, UK), and was supplemented with 3% (*w*/*v*) sucrose and 0.01% (*w*/*v*) myo-inositol [2]. The cultures were maintained under conditions similar to those used for germination. The cultures were kept for 6 weeks for bulking up of the plant material.

### 3.2. Influence of Cytokinins on Shoot Proliferation

An experiment to investigate the influence of CKs on shoot proliferation was conducted using nodal explants after the initial bulking up. Each 0.5 cm long nodal explant was aseptically excised within a laminar flow hood and cultured in the same jars utilized during the bulking up phase. Each culture jar contained a single explant, with or without CKs. Adopting a completely randomized design, three types of CKs-*m*T, BA and Kin, were individually added to the MS medium at four concentrations: 1.0, 5.0, 10.0, and 15.0 µM. The selected concentration range was based on previous studies in *Lamiaceae* and other medicinal plant species, where optimal shoot proliferation was frequently observed within this range (e.g., *M. officinalis*, *Salvia sclarea*, *Salix tetrasperma*) [26,33,60,61]. Additionally, a preliminary side experiment was conducted using lower concentrations (<1 µM), but these treatments yielded suboptimal results and were therefore excluded from the main study.

Each medium contained only one CK concentration, and a control treatment without CKs was included for comparison. Kin and BA were sourced from Thermo Fisher Scientific (Heysham, UK), while *m*T was obtained from the Laboratory of Growth Regulators, Palacky University and Institute of Experimental Botany, Olomouc, Czech Republic. Each treatment was replicated 15 times, a sample size selected based on established protocols in plant tissue culture to ensure statistical reliability and reproducibility while maintaining practical feasibility. The cultures were maintained under identical conditions as during germination. After 6 weeks of incubation, measurements of shoot and root numbers as well as shoot height, were recorded for each explant.

### 3.3. Influence of the Interaction of mT and Indole-3-Butyric Acid on Shoot Proliferation

Building on the results of the previous experiment, nodal explants cultured on medium without PGRs were transferred to similar glass jars containing either 1 µM or 0.5 µM *m*T, combined factorially with different concentrations (0, 1.0, 3.0, and 5.0 µM) of IBA. The IBA was obtained from Duchefa Biochemie (Haarlem, The Netherlands). Each treatment included 15 replicates. The cultures were maintained under identical conditions as the previous experiment. After 6 weeks of incubation, data on the number of shoots and roots as well as height of each shoot were recorded for each explant.

### 3.4. In Vitro Root Induction and Subsequent Acclimatization

Building on the previous experimental findings, multiple shoots from the treatment with 1 µM *m*T were carefully separated under aseptic conditions within a laminar flow hood. These shoots were then subcultured into similar glass culture jars, each containing 30 mL of either full-strength MS medium or sterile half-strength MS medium with 0.005% (*w*/*v*) myo-inositol and 1.5% (*w*/*v*) sucrose, both devoid of PGRs. The cultures were maintained under the same conditions as before for a duration of 8 weeks. After this incubation period, the number of roots and shoots per explant were recorded non-destructively, meaning the explants were left intact and not harvested during measurement.

The rooted plantlets from this stage were carefully washed with running tap water to remove residual agar, then transplanted into 25 cm pots containing a mixture of hygromix (Hygrotech, Pretoria, South Africa) and river sand in a 1:1 ratio (*v*/*v*). Hygromix is a growth medium composed of peat moss with added nutrients, vermiculite and polystyrene. These were subsequently transferred to a glasshouse fitted with a misting system. Based on preliminary trials, the plants were misted for 48 h to promote high humidity, and subsequently misting was maintained for 7 h each day. After 5 weeks in the glasshouse, data on survival rate and plant height were collected.

### 3.5. Statistical Analysis

One way analysis of variance (ANOVA) was performed using Statsoft (Statistica 8). To distinguish between treatment mean values when significant differences were found, Duncan’s multiple range test (*p* = 0.05) was applied. The results were presented as the mean ± standard error for each treatment.

## 4. Conclusions

This study demonstrated that *m*T is an effective and sustainable alternative to traditional CKs for in vitro propagation of *T*. *vulgaris*. The optimal shoot proliferation was achieved with 1 μM *m*T treatment, while rooting in PGR-free medium was not significantly different from that with some tested PGRs, making PGR-free medium the most economical choice for rooting. Additionally, shoots rooted in half-strength MS medium exhibited superior growth compared to those rooted in full-strength MS medium, highlighting the benefit of reduced mineral concentrations. The regenerated plants achieved 100% acclimatization, indicating the robustness and reliability of the micropropagation protocol.

From an ecological and economic standpoint, the use of PGR-free rooting medium is likely to reduce costs and chemical load, as it minimizes reliance on synthetic PGRs and lowers the risk of residual compounds in regenerated plants. For example, media supplemented with CKs and auxins can cost up to 30–40% more per 100 explants compared to hormone-free formulations, depending on the concentration and supplier. Moreover, optimizing medium strength by using half-strength MS further contributes to cost-efficiency and supports large-scale production. The findings suggest that *m*T can serve as a replacement CK thereby improving the overall efficiency, sustainability, and cost-effectiveness of the in vitro propagation protocols for *T*. *vulgaris*. This approach has significant implications for commercial cultivation, conservation efforts, and the sustainable production of thyme, aligning with modern agricultural and ecological standards.

While this study demonstrates the effectiveness of *m*T in enhancing shoot proliferation and rooting in *T. vulgaris*, certain limitations remain. The study was conducted on a single genotype under controlled conditions, and responses may vary across different cultivars or environmental setups. Additionally, the long-term genetic stability and secondary metabolite profiles of the regenerated plants were not assessed, which are critical for commercial and medicinal applications. Future studies should explore these aspects and evaluate the performance of *m*T under varying culture conditions and across diverse thyme genotypes.

## Figures and Tables

**Table 1:** Effect of full-strength and half-strength Murashige and Skoog (MS) medium on rooting and shoot proliferation.

Medium	Mean Root Number	Mean Shoot Number
Full-strength MS	7.14 ± 0.39	6.00 ± 0.37
Half-strength MS	10.75 ± 0.44 *	8.43 ± 0.54 *

Values are mean ± standard error, *n* = 15. * Statistical significance (*p* = 0.05) between the two treatments.

**Table 2:** Acclimatization and growth of regenerated *Thymus vulgaris* plantlets rooted on plant growth-free Murashige and Skoog (MS) medium after 5 weeks of culturing.

Medium	No of Plants Potted	Survival Rate (%)	Mean Plant Height (cm)
Full-strength MS	28	100	13.39 ± 0.53
Half-strength MS	28	100	14.93 ± 0.91

Values are mean ± standard error, *n* = 28. Statistical comparison between the two treatments was performed using ANOVA; significance was determined at *p* = 0.05.

## Data Availability

The data presented in this study is available on request from the corresponding author.

## Supplementary Materials

The following supporting information can be downloaded at: https://www.mdpi.com/article/10.3390/plants14233567/s1. Figure S1. Rooting of regenerated *Thymus vulgaris* shoots on (a) full-strength and (b) half-strength Murashige and Skoog medium after 8 weeks of culturing.

## Abbreviations

The following abbreviations are used in this manuscript:

2iP	6-(γ,γ-Dimethylallylamino)purine
BA	6-Benzyladenine
CK	Cytokinin
DMRT	Duncan’s multiple range test
IBA	Indole-3-butyric acid
Kin	Kinetin
MS	Murashige and Skoog
*m*T	*Meta*-topolin
PGR	Plant growth regulator
PTC	Plant tissue culture
TDZ	Thidiazuron
