# Supplementary material for: Meta-Topolin as an Aromatic Cytokinin for In Vitro Propagation of Thymus vulgaris L
Source: Plants (Basel). 2025 Nov 22;14(23):3567. doi: 10.3390/plants14233567 (PMC12693787; doi:10.3390/plants14233567)
Supplement: Supplementary file 1 [file plants-14-03567-s001.zip › plants-3998112-supplementary.pdf]

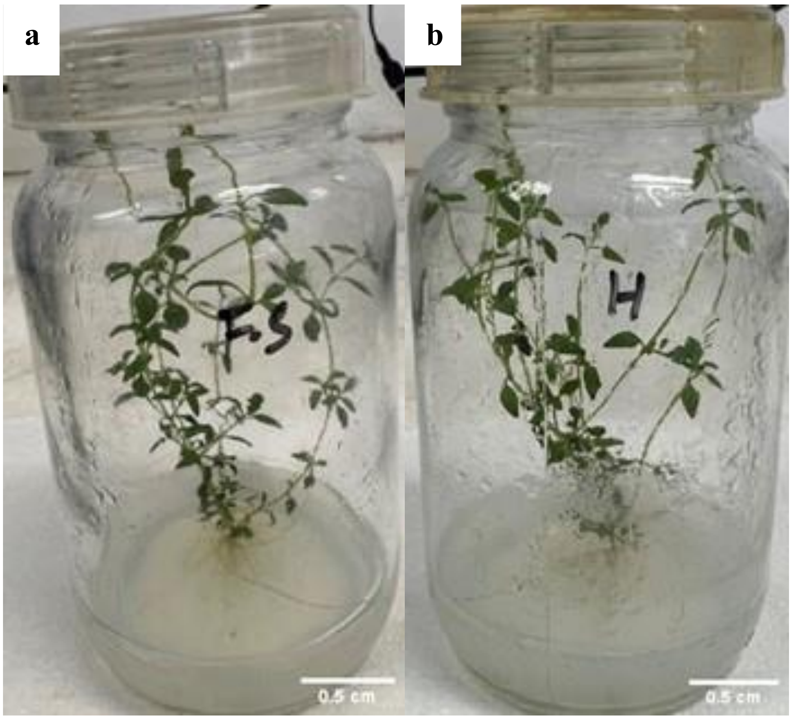

Figure S1. Rooting of regenerated *Thymus vulgaris* shoots on (**a)** full strength and **(b)** half strength Murashige and Skoog medium after 8 weeks of culturing.
